# DUSP10 Negatively Regulates the Inflammatory Response to Rhinovirus through Interleukin-1β Signaling

**DOI:** 10.1128/JVI.01659-18

**Published:** 2019-01-04

**Authors:** Grace C. A. Manley, Clare A. Stokes, Elizabeth K. Marsh, Ian Sabroe, Lisa C. Parker

**Affiliations:** a Department of Infection, Immunity and Cardiovascular Disease, University of Sheffield, Sheffield, United Kingdom; University of Texas Southwestern Medical Center

**Keywords:** COPD, IL-1β, asthma/allergy, epithelial cells, inflammation, lung infection, rhinovirus

## Abstract

Rhinoviral infection is a common trigger of the excessive inflammation observed during exacerbations of asthma and chronic obstructive pulmonary disease. Rhinovirus (RV) recognition by pattern recognition receptors activates the mitogen-activated protein kinase (MAPK) pathways, which are common inducers of inflammatory gene production. A family of dual-specificity phosphatases (DUSPs) can regulate MAPK function, but their roles in rhinoviral infection are not known. We hypothesized that DUSPs would negatively regulate the inflammatory response to RV infection. Our results revealed that the p38 and c-Jun N-terminal kinase (JNK) MAPKs play key roles in the inflammatory response of epithelial cells to RV infection. Three DUSPs previously shown to have roles in innate immunity (DUSPs 1, 4, and 10) were expressed in primary bronchial epithelial cells, and one of them, DUSP10, was downregulated by RV infection. Small interfering RNA-mediated knockdown of DUSP10 identified a role for the protein in negatively regulating inflammatory cytokine production in response to interleukin-1β (IL-1β), alone and in combination with RV, without any effect on RV replication. This study identifies DUSP10 as an important regulator of airway inflammation in respiratory viral infection.

**IMPORTANCE** Rhinoviruses are one of the causes of the common cold. In patients with asthma or chronic obstructive pulmonary disease, viral infections, including those with rhinovirus, are the commonest cause of exacerbations. Novel therapeutics to limit viral inflammation are clearly required. The work presented here identifies DUSP10 as an important protein involved in limiting the inflammatory response in the airway without affecting immune control of the virus.

## Abstract (precis)

Rhinoviruses are one of the causes of the common cold. In patients with asthma or chronic obstructive pulmonary disease, viral infections, including those with rhinovirus, are the commonest cause of exacerbations. Novel therapeutics to limit viral inflammation are clearly required. The work presented here identifies DUSP10 as an important protein involved in limiting the inflammatory response in the airway without affecting immune control of the virus.

## INTRODUCTION

Human rhinoviruses (RV) frequently trigger exacerbations of airway diseases, such as asthma and chronic obstructive pulmonary disease (COPD), where excessive inflammation causes worsening airway obstruction and increased symptoms. RV belong to the *Picornaviridae* family of viruses, which are composed of positive-sense single-stranded RNA packaged into icosahedral virions. There are over 150 serotypes, classified either phylogenetically (groups A, B, and C) or based on the receptor to which the virus binds on the cell surface (1, 2). The major group viruses, comprising most of group A and all of group B, bind intracellular adhesion molecule-1 (3), and the minor group viruses, comprising the remainder of group A, bind low-density lipoprotein receptor or related proteins (4, 5). Culture methods for group C viruses were discovered relatively recently, so investigation of this group has been limited; however, these viruses are known to bind cadherin-related family member 3 (6, 7).

RV infect airway epithelial cells, which express several pattern recognition receptors capable of recognizing distinct parts of the virus (8). Toll-like receptor 3 (TLR3) and the RIG-like receptors (RLRs) bind double-stranded RNA replication intermediates, and TLR2 on the cell surface binds the rhinoviral capsid (9–12). It has been suggested that TLRs 7 and 8 may also contribute to the response to RV (13, 14), but we and others have found airway epithelial cells to be unresponsive to TLR7/8 ligands (10, 15–17). Activation of pattern recognition receptors leads to the production and release of inflammatory cytokines through several pathways, including the NF-κB, interferon regulatory factor (IRF), and mitogen-activated protein kinase (MAPK) pathways. The p38, c-Jun N-terminal kinase (JNK), and extracellular signal-regulated kinase (ERK) MAPK pathways consist of a three-tier kinase cascade culminating in phosphorylation of the MAPK on two residues: tyrosine and threonine. The activated MAPKs translocate into the nucleus and activate a range of transcription factors, including AP-1, ATF, CREB, c/EBP, and NF-κB. The p38 pathway can also be activated by binding and internalization of RV (18–20). Previous work has shown the importance of p38 and ERK MAPKs in inducing cytokine release in response to RV infection of airway epithelial cell lines (21–23). This inflammatory response to RV can be potentiated further by interleukin-1β (IL-1β). IL-1β signals through pathways similar to those of the TLRs and is known to activate the MAPKs (24, 25). Furthermore, IL-1β is released from RV-infected immune cells, such as monocytes and macrophages, and would therefore be present in the infected airway (11). Previous work by our group showed the importance of IL-1β in the immune response to RV: RV infection induces the release of both IL-1α and IL-1β, while blocking IL-1 signaling significantly inhibits proinflammatory cytokine release (26). Furthermore, the addition of IL-1β enhances cytokine production from epithelial cells in response to RV infection (27). Thus, it is imperative that the MAPK pathways be regulated in order to stop overproduction of cytokines and excessive inflammation.

Dual-specificity phosphatases (DUSPs) are a family of proteins capable of dephosphorylating two residues in one substrate simultaneously. A subgroup of DUSPs, MAPK phosphatases (MKPs), dephosphorylate the MAPK proteins directly. Ten MKPs have been characterized so far, and three (DUSP1 [MKP1], DUSP4 [MKP2], and DUSP10 [MKP5]) have been shown to negatively regulate innate immune signaling. Knockout mice which lack each of these proteins individually produce higher levels of inflammatory cytokines in response to TLR4 activation, associated with increased p38 and/or JNK MAPK activation (28–32). It should be noted that another group has shown a conflicting role for DUSP4, with knockout mice producing lower levels of cytokines in response to TLR4 signaling (33). Much of this work has explored the roles of DUSPs in bacterial infection, and little is known about the ability of DUSPs to regulate the response to viruses, particularly within epithelial cells. More recently, bone marrow-derived macrophages (BMDMs) and dendritic cells taken from DUSP10 knockout mice were shown to exhibit increased release of inflammatory cytokines and antiviral interferons (IFNs) in response to influenza virus infection (34).

We hypothesized that one or more DUSPs play a critical role in regulating the inflammatory response to RV infection. We determined that the p38 and JNK pathways were responsible for a large proportion of the CXCL8 produced by primary bronchial epithelial cells (PBECs) in response to RV infection, while ERK did not play as great a role. DUSPs 1, 4, and 10 were expressed by PBECs. Expression of DUSPs 1 and 4 was unaltered by RV infection or IL-1β stimulation; however, RV decreased expression of DUSP10. Small interfering RNA (siRNA)-mediated knockdown of DUSP10 identified a role for the protein in regulating the response to IL-1β, both alone and in combination with RV. These results identify DUSP10 as an important regulator of the inflammatory response in epithelial cells and therefore a potential future therapeutic target for RV-induced acute exacerbations.

## RESULTS

### The p38 and JNK pathways play important roles in cytokine production in response to RV.

It is well documented that the MAPK pathways play roles in inducing cytokine release in response to a variety of stimulants. This was previously demonstrated for p38 and ERK in response to RV infection, with inhibition of either decreasing the release of CXCL8, a neutrophil chemoattractant (21–23). However, these studies utilized airway epithelial cell lines (BEAS-2B and 16HBE14o-), and the roles of these pathways in the response of PBECs to RV are not well characterized. In addition, the role of JNK in the response to RV is unknown, although this MAPK has been shown to be critical in inducing CXCL8 release in a human astroglioma cell line in response to poly(I:C), a synthetic TLR3 ligand (35).

**FIG 1:**
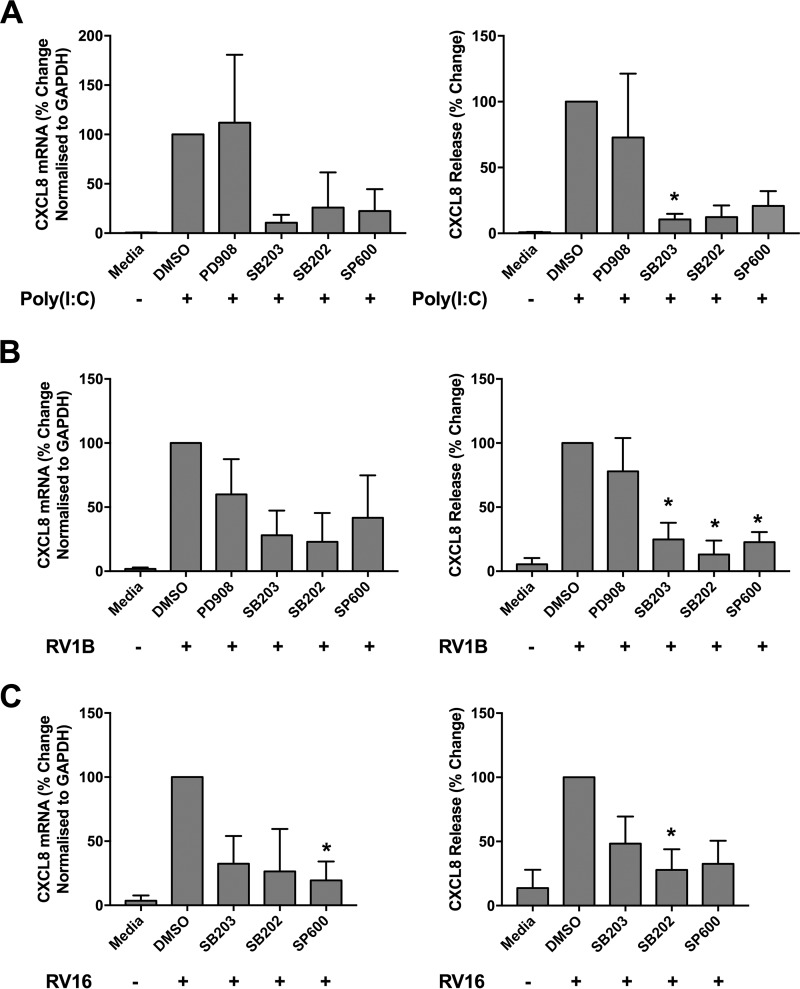
Inhibition of the p38 or JNK pathway reduces cytokine release in response to RV infection. PBECs were treated with medium only, DMSO, or a MAPK inhibitor (20 μM) (PD90859 [ERK], SB203580 [p38], SB202190 [p38], or SP600125 [JNK]) for 1 h prior to stimulation with poly(I:C) (25 μg/ml) for 24 h (*n* = 3) (A) or infection with RV1B (MOI = 3) (B) or RV16 (MOI = 4) (C) for 48 h (*n* = 4 individual donors). Inhibitors remained present throughout the experiment. Supernatants and cell lysates were collected, and levels of CXCL8 mRNA and release were measured by qRT-PCR and ELISA. Data shown are means ± SEM and are normalized to data for RV-plus-DMSO-treated cells. The significance of differences versus RV-plus-DMSO-treated cells, as measured by one-way analysis of variance (ANOVA) and Dunnett’s posttest on log raw data, is indicated as follows: *, *P* ≤ 0.05; and **, *P* ≤ 0.01.

To explore the contribution of each MAPK pathway to cytokine production in response to viral infection, we used a panel of MAPK inhibitors. PBECs were pretreated with the inhibitors for 1 h prior to stimulation with a synthetic double-stranded RNA viral mimic, poly(I:C), and inhibitors remained present throughout the 24-h stimulation. Production of the inflammatory cytokine CXCL8, a downstream target of NF-κB activation, was measured at both the RNA and protein secretion levels by use of quantitative reverse transcription-PCR (qRT-PCR) and enzyme-linked immunosorbent assay (ELISA). Poly(I:C) stimulation led to an upregulation of CXCL8 mRNA expression and protein release which was unaffected by inhibition of ERK with PD90859. Inhibition of p38 or JNK reduced CXCL8 levels; however, this was statistically significant at the protein level only for SB203580 (Fig. 1A). A similar pattern was observed in response to infection with major and minor group strains of rhinovirus, i.e., RV16 and RV1B, respectively. PBECs were treated with MAPK inhibitors for 1 h prior to infection with RV; the inhibitors were present for the 1-h RV infection and remained present for the following 48 h. CXCL8 expression was measured at 48 h, as peak cytokine release was observed at this time point (data not shown). Infection of PBECs with RV induced an increase in CXCL8 mRNA and protein secretion. CXCL8 levels were dramatically reduced by inhibition of p38 with either SB203580 or SB202190 or of JNK with SP600125, although the reductions were less clear at the mRNA level (Fig. 1B and C). Inhibition of ERK by PD90859 did not significantly affect CXCL8 expression or release in response to RV1B infection (Fig. 1B). These data suggest that p38 and JNK have important roles in inducing CXCL8 production in response to infection with major or minor strains of RV, while ERK plays a lesser role.

### DUSPs are expressed by PBECs.

**FIG 2:**
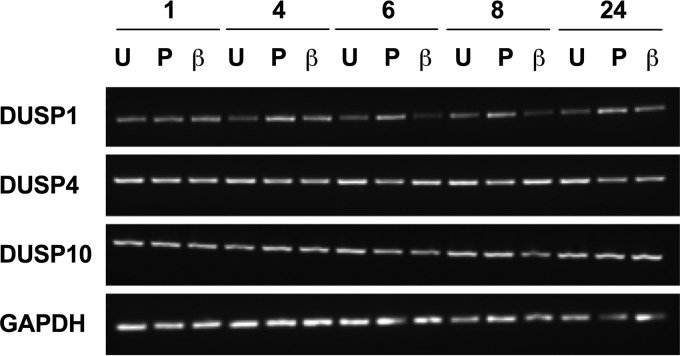
DUSPs 1, 4, and 10 are expressed in PBECs. PBECs were stimulated with poly(I:C) (25 μg/ml) (P) or IL-1β (10 ng/ml) (β) or left untreated (U) for 24 h. mRNA was collected at the indicated time points (hours), and RT-PCR was performed using primers for DUSPs 1, 4, and 10 and a GAPDH control. A representative gel is shown (*n* = 2 individual donors).

We therefore went on to investigate the expression and roles of DUSPs, important regulators of the MAPK pathways. To the best of our knowledge, the expression of DUSPs in PBECs has not previously been characterized. We first determined the gene expression of DUSPs 1, 4, and 10 by using RT-PCR. Each DUSP was expressed by PBECs, even in unstimulated cells (Fig. 2). The regulation of this expression in response to poly(I:C) or IL-1β stimulation was examined over 24 h; however, no clear changes in the expression of any of the DUSPs examined were observed using this method (Fig. 2).

**FIG 3:**
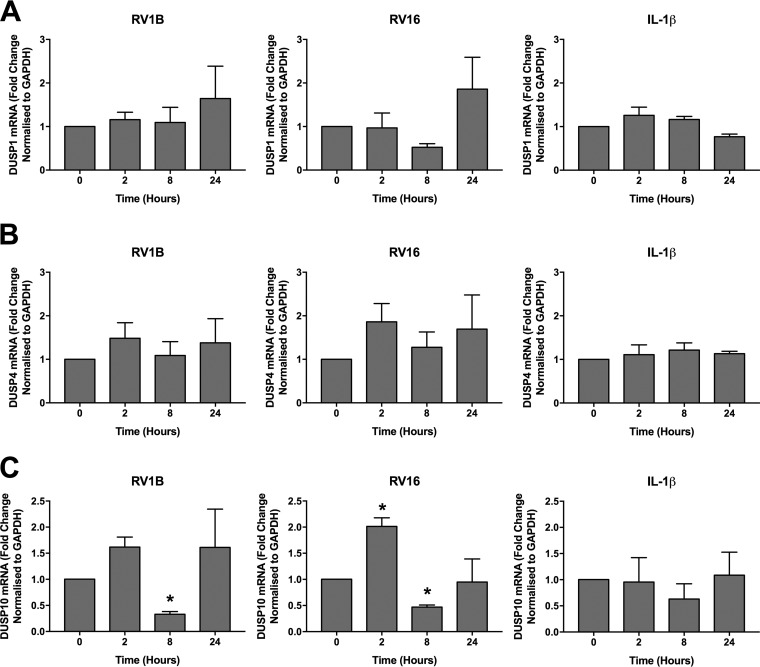
DUSP10 expression is decreased by RV infection. PBECs were infected with RV1B (MOI = 3) or RV16 (MOI = 4) or treated with IL-1β (100 ng/ml) for 24 h, and cell lysates were collected at the indicated time points. DUSP1 (A), DUSP4 (B), and DUSP10 (C) expression was measured using qRT-PCR. Data shown are means ± SEM for 3 individual donors. The significance of differences versus uninfected control cells (0 h), as measured by one-way ANOVA and Dunnett’s posttest on log Δ*C_T_* values, is indicated as follows: *, *P* ≤ 0.05.

In order to examine DUSP1, -4, and -10 mRNA expression in more detail, we utilized a more sensitive technique, namely, qRT-PCR. PBECs were infected with RV1B or RV16 or stimulated with IL-1β for 24 h, and qRT-PCR was used to measure expression of DUSPs 1, 4, and 10. As no change in DUSP expression was seen in response to 10 ng/ml IL-1β (Fig. 2), the concentration was increased to 100 ng/ml. Stimulation with 100 ng/ml IL-1β did not alter expression of any of the DUSPs (Fig. 3). Expression of DUSP1 and DUSP4 was unaltered by infection with RV1B (Fig. 3A and B). Infection with RV16 increased DUSP1 mRNA expression at 24 h; however, this was variable and nonsignificant (Fig. 3A). Poly(I:C) stimulation was also found to increase DUSP1 mRNA expression, as found previously (36), but had no effect on expression of DUSPs 4 and 10 (data not shown). Infection with either strain of RV caused a similar regulation of DUSP10 expression, with an initial increase followed by a consistent and significant downregulation at 8 h postinfection before a return to baseline by 24 h (Fig. 3C).

**FIG 4:**
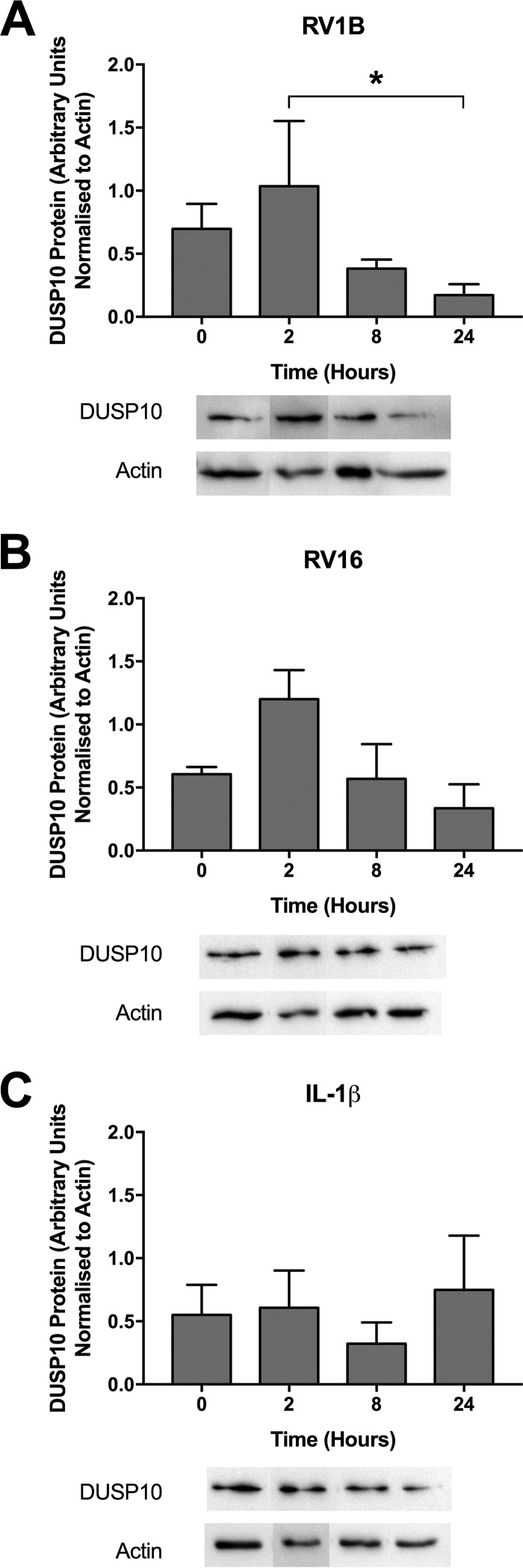
DUSP10 protein expression is decreased by RV infection. PBECs were infected with RV1B (MOI = 3) (A) or RV16 (MOI = 4) (B) or treated with IL-1β (100 ng/ml) (C) for 24 h, and cell lysates were collected at the indicated time points. DUSP10 and actin expression was measured by Western blotting. Data shown are means ± SEM for densitometry analysis of cells from 3 individual donors, with representative blots shown below the graphs. The significance of differences, as measured by one-way ANOVA and Dunnett’s posttest on log values, is indicated as follows: *, *P* ≤ 0.05. Note that blots shown are segments of longer time courses.

DUSP10 protein expression followed a similar pattern, with a slight increase 2 h following RV1B infection before a decline to a level below the baseline (Fig. 4A). RV16 infection had a similar effect on DUSP10 protein levels, but it was not statistically significant (Fig. 4B). As in the mRNA expression studies (Fig. 3), IL-1β stimulation had no effect on DUSP10 protein expression (Fig. 4C).

### DUSP10 does not regulate the response to RV.

**FIG 5:**
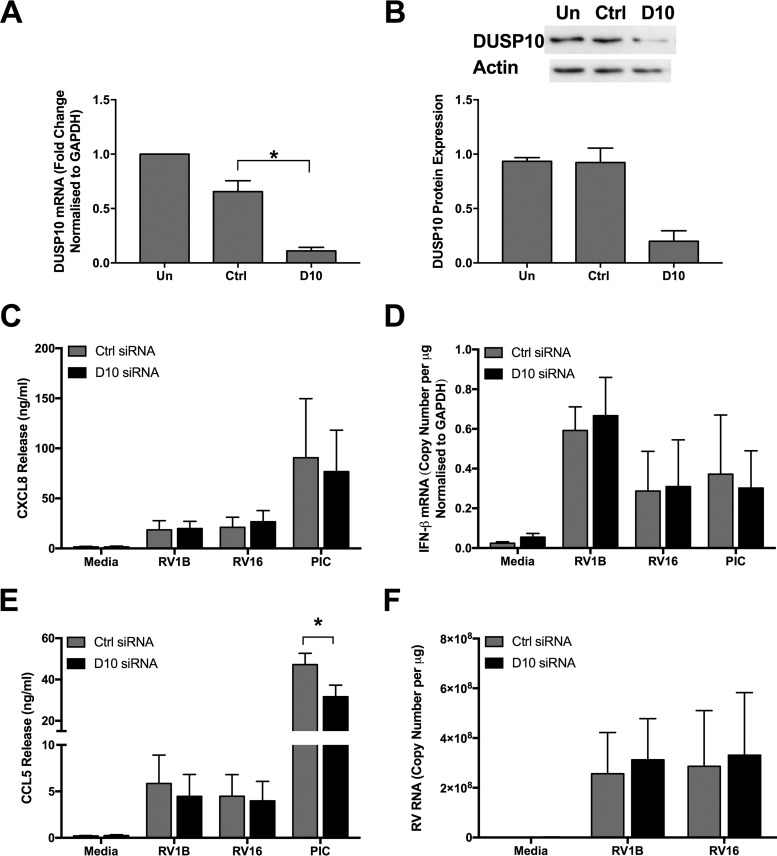
DUSP10 knockdown does not affect the response of PBECs to RV infection. PBECs were left untransfected or treated with DUSP10 (D10) or control (Ctrl) siRNA (100 nM) for 48 h. Cell lysates and supernatants were collected and analyzed for DUSP10 expression by qRT-PCR (A) and Western blotting (B). PBECs were then infected with RV1B (MOI = 3) or RV16 (MOI = 4) or treated with poly(I:C) (25 μg/ml). Cell lysates and supernatants were collected after 24 h and analyzed by ELISA for CXCL8 (C) and CCL5 (E) or after 16 h and analyzed by qRT-PCR for IFN-β (D). (F) RV RNA levels after 24 h were measured using qRT-PCR. Data shown are means ± SEM for 3 individual donors (A to E) or 4 individual donors (F). The significance of differences between siRNA treatments, as measured using one-way ANOVA and Dunnett’s posttest on log Δ*C_T_* values (A) or log protein expression values (B) or using two-way ANOVA and Sidak’s posttest on log data (C to F), is indicated as follows: *, *P* ≤ 0.05.

Among the proteins examined, DUSP10 was the only one found to be regulated by RV infection and thus was chosen for further investigation. siRNA was used to successfully knock down DUSP10 expression in PBECs, reducing DUSP10 mRNA and protein levels to approximately 20% of control levels (Fig. 5A and B). Control and DUSP10 knockdown cells were then infected with RV1B or RV16 or stimulated with poly(I:C) for 24 h, and the release of the inflammatory protein CXCL8 was measured by ELISA. Release of CXCL8 was unaffected by DUSP10 knockdown (Fig. 5C).

As DUSP10 was previously shown to regulate type I IFN production in response to influenza virus infection (34), the level of IFN-β mRNA was measured at 16 h post-RV infection. Low levels of IFN-β were detected by qRT-PCR in response to poly(I:C) or either strain of RV, and this was unaffected by DUSP10 knockdown (Fig. 5D). DUSP10 knockdown also had no effect on levels of release of the interferon-stimulated gene CCL5 in response to RV (Fig. 5E). In response to poly(I:C) stimulation, CCL5 levels were reduced by DUSP10 knockdown. However, this may have been due to cell death caused by DUSP10 knockdown, as observed by eye (data not shown). RV replication at 24 h was also unaffected by DUSP10 knockdown, with RV RNA levels being similar for control and DUSP10 siRNA treatments (Fig. 5F).

### DUSP10 regulates the response to IL-1β.

**FIG 6:**
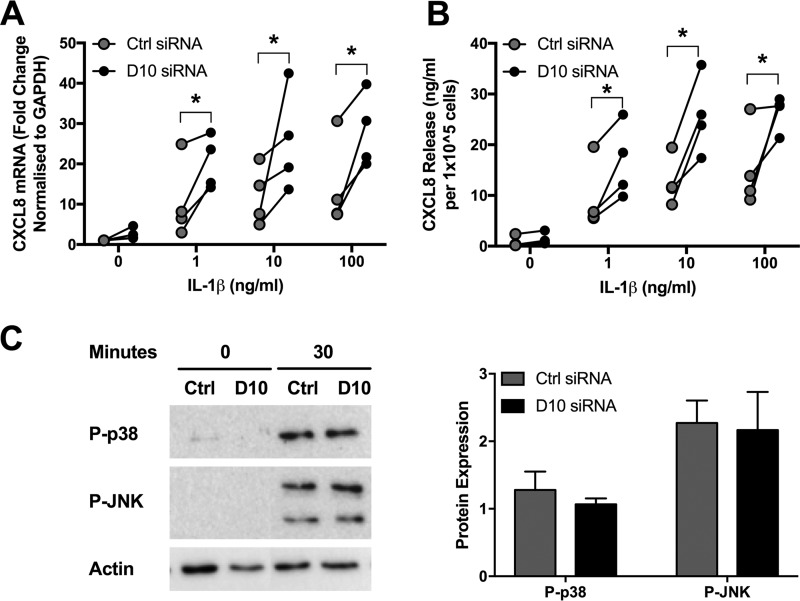
DUSP10 knockdown increases cytokine production but not MAPK activation in response to IL-1β. PBECs were treated with DUSP10 (D10) or control (Ctrl) siRNA (100 nM) for 48 h prior to stimulation with IL-1β (1 to 100 ng/ml) for 24 h. Cell lysates and supernatants were collected and CXCL8 measured by qRT-PCR (A) and ELISA (B) for 4 individual donors. (C) PBECs were treated with siRNA for 48 h prior to stimulation with IL-1β (100 ng/ml) for 30 min. Cell lysates were collected, and levels of phosphorylated p38 and JNK and total actin were measured using Western blotting. Data shown are means ± SEM of band densities for 3 individual donors, and representative blots are shown. The significance of differences between siRNA treatments, as measured by two-way ANOVA and Sidak’s posttest on log data, is indicated as follows: *, *P* ≤ 0.05.

While DUSP10 knockdown did not affect the response of PBECs to RV, the response to IL-1β was altered. Stimulation of PBECs with a range of IL-1β concentrations induced mRNA production and protein release of CXCL8 (Fig. 6A and B). CXCL8 mRNA and protein levels were significantly increased in cells with reduced DUSP10 levels (Fig. 6A and B). As p38 and JNK were shown to be important inducers of CXCL8 production, the effect of DUSP10 knockdown on IL-1β-induced MAPK activation was investigated. Levels of phosphorylated, activated p38 and JNK in response to IL-1β were measured in cells treated with DUSP10 or control siRNA. IL-1β stimulation upregulated phosphorylation of both proteins, but the level of activation was unaffected by DUSP10 knockdown (Fig. 6C).

**FIG 7:**
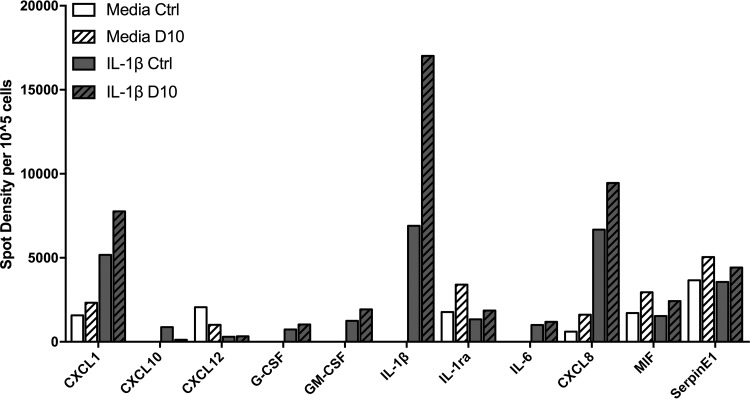
Effect of DUSP10 knockdown on cytokine expression in response to IL-1β. PBECs were treated with DUSP10 (D10) or control (Ctrl) siRNA (100 nM) for 48 h prior to stimulation with IL-1β (10 ng/ml) for 24 h. Supernatants were collected and cytokine array analysis performed. Data presented are spot densities normalized to cell number (*n* = 1).

In order to gain a wider view of the role of DUSP10 in IL-1β signaling, an array was used to determine the effect of DUSP10 knockdown on the release of a variety of cytokines. The chosen array contained antibodies specific for 36 proteins known to be upregulated in response to inflammation (ARY005B; R&D Systems). The level of each protein released by cells from one donor that were treated with DUSP10 or control siRNA prior to 24 h of stimulation with IL-1β was determined. IL-1β stimulation increased release of several cytokines by PBECs, including CXCL1, CXCL10, granulocyte colony-stimulating factor (G-CSF), granulocyte-macrophage colony-stimulating factor (GM-CSF), IL-6, CXCL8, and IL-1β itself, and decreased the release of CXCL12 (Fig. 7). In keeping with the data shown above, DUSP10 knockdown potentiated the IL-1β-induced release of CXCL1, CXCL8, and IL-1β, with IL-1β levels increasing 1.71-fold compared to those in cells treated with control siRNA (Fig. 7). Intriguingly, DUSP10 knockdown decreased levels of CXCL10 release in response to IL-1β. These data supported a role for DUSP10 in regulating the inflammatory response of airway epithelial cells.

### IL-1β is released by PBECs in response to RV infection.

**FIG 8:**
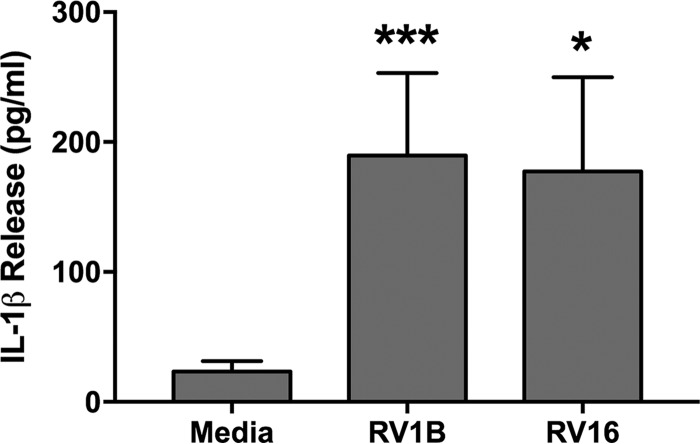
IL-1β is released in response to RV infection. PBECs were infected with RV1B (MOI = 3) or RV16 (MOI = 4) for 24 h. Supernatants were collected, and levels of IL-1β release were measured by ELISA. Data shown are means ± SEM for 3 individual donors. The significance of differences versus uninfected cells, as measured by one-way ANOVA and Dunnett’s posttest on log data, is indicated as follows: *, *P* ≤ 0.05; and ***, *P* ≤ 0.001.

In order to determine whether the role of DUSP10 in the response to IL-1β has relevance to RV infection, the release of IL-1β in response to RV was quantified. PBECs released around 180 pg/ml IL-1β in response to 24 h of infection with either RV1B or RV16 (Fig. 8).

### DUSP10 regulates the response of PBECs to dual stimulation with RV and IL-1β.

**FIG 9:**
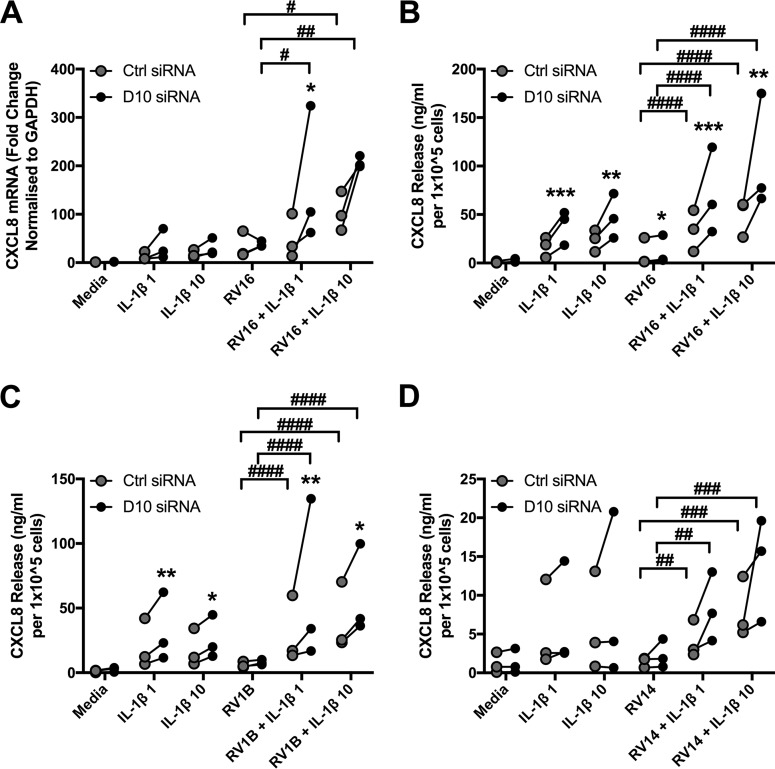
IL-1β potentiates the response of PBECs to RV infection. PBECs were treated with control (Ctrl) or DUSP10 (D10) siRNA (100 nM) for 48 h prior to stimulation with IL-1β (1 or 10 ng/ml) and/or infection with RV16 (MOI = 4) (A and B), RV1B (MOI = 3) (C), or RV14 (D) for 24 h. Cell supernatants and lysates were collected and CXCL8 measured by qRT-PCR (A) and ELISA (B to D). The significance of differences between Ctrl and D10 siRNAs, as measured by two-way ANOVA and Sidak’s posttest, is indicated as follows: *, *P* ≤ 0.05; **, *P* ≤ 0.01; ***, *P* ≤ 0.001; and ****, *P* ≤ 0.0001 (*n* = 3 individual donors). The significance of differences between RV alone and RV in combination with IL-1β, as measured by two-way ANOVA and Dunnett’s posttest on log raw data, is indicated as follows: #, *P* ≤ 0.05; ##, *P* ≤ 0.01; ###, *P* ≤ 0.001; and ####, *P* ≤ 0.0001 (*n* = 3 individual donors).

IL-1β is an important early signaling molecule in the airway. It was previously shown to potentiate the response of airway epithelial cells to RV infection, increasing the release of CXCL8 by the bronchial epithelial cell line BEAS-2B (27). We therefore investigated whether IL-1β would potentiate the response of PBECs to RV and determined the role of DUSP10 in this setting. Stimulation with IL-1β or infection with RV16 caused modest increases in CXCL8, while the addition of IL-1β to RV16-infected cells significantly augmented CXCL8 release. At the mRNA level, only the higher dose of IL-1β, 10 ng/ml, caused observable increases in CXCL8 production compared to that with RV16 alone (Fig. 9A). However, both concentrations caused incremental increases in CXCL8 release (Fig. 9B). This response was further potentiated by DUSP10 knockdown, with significantly higher CXCL8 mRNA and protein levels than those in control siRNA-treated cells. A similar pattern was seen in response to infection with the minor group virus RV1B (Fig. 9C). To ensure that the increased CXCL8 observed on dual stimulation was not due to an effect of IL-1β on viral replication, the intracellular viral RNA levels were quantified by qRT-PCR. No significant effects were observed between RV16 alone and the same virus in combination with IL-1β (data not shown).

In addition to the major and minor classifications of rhinoviruses, they are grouped phylogenetically, into groups A, B, and C. Both RV1B and RV16 belong to group A. Therefore, RV14, a major group rhinovirus representing a third serotype and belonging to group B, was examined. In accordance with previous results, infection of PBECs with RV14 led to a small increase in CXCL8 release which was unaffected by DUSP10 knockdown. When RV14 infection was combined with IL-1β stimulation, CXCL8 release was increased and was further potentiated by DUSP10 knockdown (Fig. 9D). These data demonstrate a role for DUSP10 in combination with IL-1β in negatively regulating the response of PBECs to RV.

### DUSP10 has a similar role in PBECs isolated from COPD patients.

**FIG 10:**
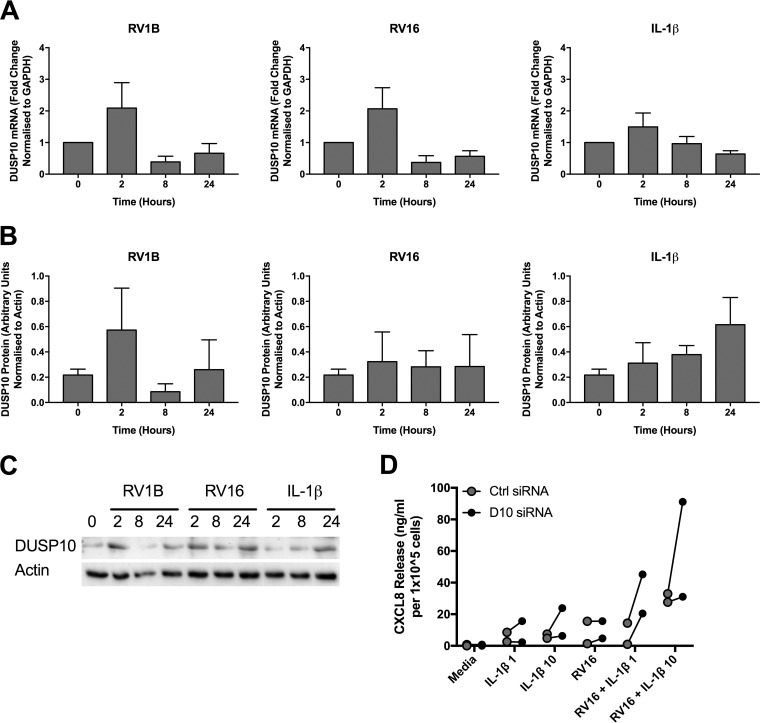
DUSP10 expression is decreased by RV infection in COPD cells, and DUSP10 knockdown increases CXCL8 release. COPD PBECs were infected with RV1B (MOI = 3) or RV16 (MOI = 4) or treated with IL-1β (100 ng/ml) for 24 h, and cell lysates were collected at the indicated time points. DUSP10 expression was measured using qRT-PCR (A) and Western blotting (B). Data shown are means ± SEM for individual donors. Densitometry results are shown in panel B, and a representative blot is shown in panel C. The significance of differences was measured by one-way ANOVA and Dunnett’s posttest on log Δ*C_T_* values (A) or log densitometric values (B). (D) COPD PBECs were treated with control (Ctrl) or DUSP10 (D10) siRNA (100 nM) for 48 h prior to stimulation with IL-1β (1 or 10 ng/ml) and/or infection with RV16 (MOI = 4) for 24 h. Cell supernatants were collected and CXCL8 measured by ELISA (*n* = 2 individual donors).

In order to ensure that the role of DUSP10 is clinically relevant, its role in PBECs isolated from chronic obstructive pulmonary disease (COPD) patients was investigated. Infection of COPD PBECs with RV1B caused a pattern of change in expression of DUSP10 mRNA and protein similar to that seen for normal PBECs, with an initial increase followed by a downregulation by 8 h (Fig. 10). RV16 infection also had an effect on DUSP10 mRNA expression similar to that seen in normal PBECs, but this was not observed at the protein level. In keeping with the observations for normal PBECs, IL-1β stimulation did not affect expression of DUSP10 at either the mRNA or protein level. Furthermore, siRNA-mediated knockdown of DUSP10 in COPD PBECs increased release of CXCL8 in response to a combination of RV16 and IL-1β stimulation, as seen in normal PBECs.

## DISCUSSION

Rhinoviral infection causes exacerbations of underlying airway disease through excessive inflammatory responses. The MAPKs are known to be activated by rhinoviral infection (18–21, 37); however, the roles of each pathway in the inflammatory response of primary cells to RV have not been well characterized. Previous studies have found that inhibition of ERK or p38 reduces production of inflammatory cytokines in response to RV (21–23). In accordance with this, inhibition of the p38 or JNK MAPK led to a decrease in inflammatory cytokine production, but ERK was found to have a lesser role, demonstrating differences between previously studied cell lines (BEAS-2B and 16HBE14o-) and primary cells. Although small-molecule inhibitors may have off-target effects (38), the results strongly indicate that p38 and JNK are important inducers of inflammation in RV infection.

Three members of the DUSP family, DUSPs 1, 4, and 10, have been shown to negatively regulate MAPK pathways in innate immune signaling, although their role in RV infection has not yet been studied. All three DUSPs were constitutively expressed by PBECs. DUSPs 1 and 4 were previously characterized as early response genes, with no constitutive expression of DUSP1 in primary human airway smooth muscle cells or of DUSP4 in mouse BMDMs or embryonic fibroblasts (33, 39, 40). In contrast, DUSP10 is constitutively present in HeLa cells and murine BMDMs and is upregulated by innate immune stimuli (34, 41). The expression of DUSPs 1 and 4 at baseline may be a specific characteristic of bronchial epithelial cells as opposed to macrophages or fibroblasts. Differentiation of PBECs in air-liquid interface cultures has been shown to alter expression of cellular proteins; however, previous gene expression arrays have not shown differences in DUSP1, -4, or -10 expression between submerged and differentiated cultures (42).

Infection with either strain of RV caused decreases in DUSP10 expression at the mRNA and protein levels which were not seen in response to IL-1β. In 2008, Proud et al. performed a gene expression array analysis of nasal scrapings after experimental RV16 infection. DUSP10 mRNA expression was unchanged at 8 and 48 h postinfection (43). However, as the downregulation observed in our study was transient, changes in DUSP10 expression may have occurred outside the two time points investigated in the study of Proud et al. This downregulation of DUSP10 may be a host- or virus-triggered response. In support of a host-mediated response, previous work has shown that DUSPs 1 and 6 are regulated by proteasomal degradation in cells treated with growth factors or carcinogens (44, 45). However, many viruses target host proteins for degradation, either utilizing host ubiquitin ligases or expressing their own (46, 47). Rhinovirus also encodes its own proteinases 2A and 3C, which have been found to degrade components of the IFN signaling pathway (48), and nonstructural protein 1 of human immunodeficiency virus has been shown to target DUSP1 for upregulation in order to limit the inflammatory response (49). The extent to which regulation of DUSP10 in this context may be a pathogen-driven manipulation of the host immune system remains to be determined.

In this study, DUSP10 expression was successfully knocked down using siRNA, allowing investigation into the role of this protein in RV infection of PBECs. Reduced DUSP10 levels did not affect RV replication or IFN-β production in response to RV or poly(I:C). This contrasts with the results of a study by James et al. in which influenza virus replication was decreased in DUSP10 knockout mice due to increased IFN levels (34). This implies specific roles for DUSP10 in individual pathogenic infections, potentially consequent upon differential TLR signaling by each virus (50). Interestingly, DUSP10 knockout BMDMs produced increased mRNA and secreted protein levels of IFN-β in response to poly(I:C) (34). Thus, DUSP10’s roles may be species and/or cell type specific, emphasizing the need for studies such as ours to examine its role in primary human airway epithelial cells.

Knockdown of DUSP10 did not affect cytokine release in response to RV infection or poly(I:C) stimulation. However, in response to IL-1β, DUSP10 knockdown consistently caused an increase in CXCL8 production. In order to gain a wider view of the role of DUSP10, we utilized a cytokine array. Although this technique is semiquantitative and included samples from only one donor, it gives an indication of the points at which DUSP10 may act. Interestingly, DUSP10 knockdown increased release of the neutrophil chemoattractants CXCL8 and CXCL1 and decreased release of CXCL10, a Th1 cell chemoattractant. The MAPK pathways were previously shown to downregulate CXCL10 production in response to RV16 by negatively regulating IRF1 activity (51). However, p38 and JNK MAPK activation levels were unchanged by DUSP10 knockdown, suggesting a potential novel target of DUSP10. Expression of IL-1β itself was also increased by DUSP10 knockdown, which may point toward a role for DUSP10 in inflammasome regulation. Rhinoviral infection of PBECs is known to activate the NLRP3 and NLRC5 inflammasomes, leading to IL-1β release (26, 52). More recently, RV infection was found to increase caspase 1 expression to a greater extent in asthmatic PBECs than in normal cells, and in a house dust mite murine model of asthma exacerbations, caspase 1 knockout mice had reduced Th2 responses to poly(I:C) (53). Thus, a potential role of DUSP10 in regulating the inflammasome has significant implications for asthma.

IL-1β is an important inflammatory molecule shown to have roles in asthma and COPD (54, 55), and IL-1β knockout mice have reduced neutrophilic and Th2 responses in a murine asthma model (56). Blocking of IL-1β signaling in PBECs decreases the release of inflammatory mediators in response to RV infection (26). Previous work by our group and others has found a key role for IL-1β in cooperative signaling between monocytes/macrophages and epithelial cells. *In vitro* coculture models have demonstrated that addition of monocytes to epithelial cells can exacerbate the inflammatory response to lipopolysaccharide unless IL-1β signaling is blocked by use of blocking antibodies or IL-1 receptor antagonist (IL-1Ra) (57–60). Monocytes have been shown to release IL-1β in response to RV infection (11), and cooperative signaling has also been demonstrated in the context of RV infection: addition of primary monocytes to BEAS-2B cells or PBECs increases the production of the inflammatory cytokines CXCL8, CCL2, and CXCL10 in response to RV (27, 61), and IL-1Ra inhibits this increased cytokine generation (27). In accordance with this, PBECs were found to release IL-1β in response to RV infection. Costimulating PBECs with RV and IL-1β was found to dramatically potentiate the response to RV alone. The response was further increased by loss of DUSP10. This was true for three serotypes of rhinovirus, including a major group A virus (RV16), a minor group A virus (RV1B), and a major group B virus (RV14). This suggests that DUSP10 has a role in the response to rhinoviral infection in the airway, with RV inducing IL-1β release by monocytes, which stimulates epithelial cells to release cytokines, regulated by DUSP10. This anti-inflammatory role for DUSP10 was also observed in two independent COPD donors, with increased CXCL8 release in response to RV and IL-1β costimulation when DUSP10 was knocked down. COPD and asthma patients have been shown to have increased baseline levels of IL-1β (54, 62, 63); thus, DUSP10 may have an increased role in a disease setting. However, this remains to be investigated, as it was not possible to directly compare PBECs from healthy and COPD donors in this study due to differences in isolation techniques. Therefore, any additional role of DUSP10 in inflammatory airway diseases remains to be investigated.

These data demonstrate a novel role for DUSP10 in negatively regulating the inflammatory response of epithelial cells to IL-1β alone and in combination with RV. This suggests that DUSP10 has an important role in regulating inflammation of the airway and identifies it as a potential future therapeutic target for exacerbations of asthma and chronic obstructive pulmonary disease.

## MATERIALS AND METHODS

### Cells and viruses.

Primary human bronchial epithelial cells (PBECs) isolated from healthy humans were purchased from Promocell (Heidelberg, Germany), and PBECs isolated from patients with COPD were purchased from Lonza (Basel, Switzerland). Cells were maintained as previously described (27), and all experiments were carried out on cells from at least three independent PBEC donors.

Human rhinovirus serotypes 1B and 16 were propagated in HeLa Ohio cells (American Type Culture Collection) in Dulbecco’s modified Eagle’s medium (DMEM) (Gibco) supplemented with 2% fetal calf serum (FCS) (Gibco), 2% HEPES (Gibco), 1% bicarbonate (Gibco), and penicillin-streptomycin (Invitrogen), as previously described (27). Human rhinovirus serotype 14 was a kind gift from MedImmune Ltd., Cambridge, United Kingdom. Viral titers were determined by 50% tissue culture infective dose (TCID_50_) assay in HeLa Ohio cells.

### Inhibitor treatment.

Prior to cell stimulation or infection, cells were treated with the indicated concentrations of MAPK inhibitors (Tocris) diluted in dimethyl sulfoxide (DMSO) for 1 h. Inhibitors remained present throughout the experiment.

### siRNA knockdown.

PBECs were grown in 12-well plates until 80% confluent. Lipofectamine 2000 (Invitrogen) and DUSP10 siRNA (sc-61048; Santa Cruz) or control siRNA (D-001810-02-05; Dharmacon) were diluted to the indicated concentrations in Opti-MEM (Gibco) and equilibrated at room temperature for 5 min before both solutions were combined and further equilibrated for 20 min. PBECs were washed in phosphate-buffered saline (PBS) and the medium replaced with fresh supplement-free airway epithelial cell basal medium, and siRNA mixtures were applied dropwise. Cells were incubated at 37°C and 5% CO_2_ for 4 h before the medium was replaced with airway epithelial cell basal medium supplemented with penicillin-streptomycin and an airway epithelial cell supplement pack, excepting bovine pituitary extract (recovery medium). Cells were incubated at 37°C and 5% CO_2_ for 48 h prior to stimulation or infection.

### RV infection of PBECs.

PBECs were seeded in 12-well plates and grown to 80 to 90% confluence. The medium was replaced with supplement-free airway epithelial cell basal medium, and the cells were incubated at 37°C and 5% CO_2_ overnight. Cells were incubated with RV at the indicated multiplicity of infection (MOI) for 1 h at 37°C and 5% CO_2_, with agitation. MOIs were selected, based on preliminary concentration-response optimization experiments, to provide equivalent levels of inflammatory cytokine release and intracellular RV RNA copies (Fig. 5C, D, and F). Virus was removed and replaced with recovery medium, and cells were incubated at 37°C and 5% CO_2_ for the indicated times. Cell-free supernatants or cell lysates were harvested and stored at −80°C.

### IL-1β or poly(I:C) stimulation of PBECs.

PBECs were seeded in 12-well plates and grown to 80 to 90% confluence. The medium was replaced with supplement-free airway epithelial cell basal medium, and the cells were incubated at 37°C and 5% CO_2_ overnight. The medium was replaced with recovery medium containing the indicated concentration of IL-1β (Peprotech) or low-molecular-weight poly(I:C) (Invivogen). Cells were incubated at 37°C and 5% CO_2_ for the indicated times. Cell-free supernatants or cell lysates were harvested and stored at −80°C.

### Cotreatment of PBECs with RV and IL-1β.

PBECs were infected with RV as described above. After removal of the virus, the medium was replaced with recovery medium containing the indicated concentration of IL-1β (Peprotech). Cells were incubated at 37°C and 5% CO_2_ for the indicated times. Cell-free supernatants or cell lysates were harvested and stored at −80°C.

### PCR.

RNA was extracted using Tri reagent (Sigma-Aldrich) according to the manufacturer’s instructions, and contaminating DNA was removed using a DNase treatment kit (Ambion). cDNA was generated from 1 μg RNA by use of a high-capacity cDNA reverse transcriptase kit (Applied Biosystems).

PCR was carried out using a GoTaq hot start polymerase kit (Promega) according to the manufacturer’s instructions, using primers specific to DUSPs 1, 4, and 10 and glyceraldehyde-3-phosphate dehydrogenase (GAPDH) (Sigma-Aldrich). The sequences of the primers were as follows: for DUSP1, GTCGTGCAGCAAACAGTCGA (F) and CGATTAGTCCTCATAAGGTA (R) (62); for DUSP4, TTCAACAGGCATCCATCCCT (F) and TGGCTTTGGGAGGGAATGAT (R); for DUSP10, ATGACCAAATGCAGCAAG (F) and GGAGCTGGAGGGAGTTGTCAC (R) (63); and for GAPDH, GGTGAAGGTCGGTGTGAAC (F) and CTCGCTCCTGGAAGATGGTG (R).

Quantitative PCR was carried out using primers and probes from Sigma-Aldrich for RV (SY150600935-024, SY150600935-025, and HA07878670-002) and IFN-β (SY150506722-061, SY150504450-060, and HA07784503-002) and primer-probe sets from Applied Biosystems for DUSP1 (Hs00610256_g1), DUSP4 (Hs01027785_m1), DUSP10 (Hs00200527_m1), CXCL8 (Hs00174103_m1), and GAPDH (Hs02758991_g1). Reaction mixtures were made using Promega GoTaq Probe qPCR master mix or Eurogentec qPCR master mix, and reactions were run using an ABI7900 Fast real-time PCR system (Applied Biosystems) (50°C for 2 min, 95°C for 10 min, and then 40 cycles of 95°C for 15 s and 60°C for 1 min). CXCL8, IFN-β, and RV were quantified against a standard curve of plasmids containing known copy numbers of target genes. CXCL8, IFN-β, DUSP1, DUSP4, and DUSP10 expression was normalized to that of GAPDH.

### ELISA.

An enzyme-linked immunosorbent assay (ELISA) was used to detect CXCL8 and CCL5 in cell-free supernatants by use of matched antibody pairs from R&D Systems. Levels of CXCL8 and CCL5 above the minimum detection level (78.125 pg/ml for CXCL8 and 156.25 pg/ml for CCL5) were quantified against a standard curve from the same plate.

### Cytokine array.

The presence of 36 proteins in cell-free supernatants was determined using an R&D Systems human cytokine array (ARY005B) according to the manufacturer’s instructions.

### Western blotting.

PBECs were lysed in buffer containing 1% Triton X and boiled for 5 min in SDS-PAGE buffer. Lysates were subjected to SDS-PAGE, and proteins were transferred to a nitrocellulose membrane. Membranes were blotted with antibodies to DUSP10 (Abcam), phosphorylated p38 (Promega), phosphorylated JNK (Cell Signaling), and actin (Sigma-Aldrich). Antibodies were detected using a horseradish peroxidase (HRP)-conjugated anti-rabbit secondary antibody (Dako). Densitometric analysis was performed using ImageJ software (version 1.50i; NIH).

### Statistical analysis.

All data presented, excluding those in Fig. 6, are means ± standard errors of the means (SEM) (where appropriate) for at least three independent experiments using PBECs from different donors. Data were analyzed by use of log data, using the statistical tests stated in the figure legends, as the data are lognormally distributed. In Fig. 1, Fig. 6A, and Fig. 9A, normalized data are presented due to variability between PBEC donors. For normalized data or ΔΔ*C_T_* qRT-PCR data, statistical tests were performed on raw data or Δ*C_T_* values, respectively.
